# Changes in leisure-time physical activity among Brazilian pregnant women: comparison between two birth cohort studies (2004 – 2015)

**DOI:** 10.1186/s12889-017-4036-y

**Published:** 2017-01-25

**Authors:** Carolina de Vargas Nunes Coll, Marlos Rodrigues Domingues, Pedro Curi Hallal, Inácio Crochemore Mohnsam da Silva, Diego Garcia Bassani, Alicia Matijasevich, Aluísio Barros, Iná S. Santos, Andréa Dâmaso Bertoldi

**Affiliations:** 1 Postgraduate Program in Epidemiology, Federal University of Pelotas, Pelotas, Brazil; 2 Postgraduate Program in Physical Education, Federal University of Pelotas, Pelotas, Brazil; 3 International Center for Equity in Health, Federal University of Pelotas, Pelotas, Brazil; 4 Centre for Global Child Health, The Hospital for Sick Children, Toronto, Canada; 5 Department of Paediatrics and Dalla Lana School of Public Health, University of Toronto, Toronto, Canada; 6 Department of Preventive Medicine, Faculty of Medicine, University of São Paulo, São Paulo, Brazil

**Keywords:** Surveillance, Exercise, Physical activity, Motor activity, Pregnancy, Recommendations, Birth cohort studies

## Abstract

**Background:**

Low levels of leisure-time physical activity (LTPA) during pregnancy have been shown in studies conducted worldwide. Surveillance is extremely important to monitor the progress of physical activity patterns over time and set goals for effective interventions to decrease inactivity among pregnant women. The aim of this study was to evaluate time changes in LTPA among Brazilian pregnant women in an 11-year period (2004–2015) by comparing data from two birth cohort studies.

**Methods:**

Two population-based birth cohort studies were carried out in the city of Pelotas, southern Brazil, in 2004 and 2015. A total of 4244 and 4271 mothers were interviewed after delivery. Weekly frequency and duration of each session of LTPA in a typical week were reported for the pre-pregnancy period and for each trimester of pregnancy. Trends in both recommended LTPA (≥150 min/week) and any LTPA (regardless of weekly amount) were analysed overtime. Changes were also calculated separately for subgroups of maternal age, schooling, family income, parity, pre-pregnancy body mass index and pre-pregnancy LTPA.

**Results:**

The proportion of women engaged in recommended levels of LTPA pre-pregnancy increased from 11.2% (95%CI 10.0–12.2) in 2004 to 15.8% (95%CI 14.6–16.9) in 2015. During pregnancy, no changes were observed over the period for the first (10.6 to 10.9%) and second (8.7 to 7.9%) trimesters, whereas there was a decrease from 3.4% (95%CI 2.9–4.0) to 2.4% (95%CI 1.9–2.8) in the last trimester. Major decreases in LTPA in the last trimester were observed among women who were younger, with intermediate to high income, high schooling, primiparous, pre-pregnancy obese and, engaged in LTPA before pregnancy. Changes in any LTPA practice followed the same patterns described for recommended LTPA.

**Conclusions:**

Despite the increase in the proportion of women engaged in LTPA before pregnancy between 2004 and 2005, LTPA levels remained stable during the first and second trimesters of pregnancy and declined during the third gestational trimester over the period. Interventions to encourage the maintenance of LTPA practice throughout pregnancy are urgently needed.

## Background

Promotion of physical activity is a global public health priority due to it’s potential to reduce the burden of non-communicable diseases and improve the health of populations [1]. However, inactivity remains highly prevalent in most countries; nearly a quarter of the world’s population does not meet the minimal physical activity recommendations [2]. In this scenario, adult women are more likely to be physically inactive compared to men and might be considered a priority group for planning strategies to increase physical activity levels [2]. Pregnancy is a period of particular concern since decreases in physical activity levels are observed during the childbearing years and may influence leisure-time physical activity (LTPA) patterns permanently over time [3, 4].

Low levels of LTPA during pregnancy are associated with complications that can influence maternal and child health such as gestational diabetes mellitus, excessive gestational weight gain, preeclampsia, depression, preterm birth, large for gestational age and increased neonatal adiposity [5–8]. Moreover, LTPA promotion could reduce the risk of long-term chronic diseases in the pregnant women [9] and the offspring [10].

Most of the current guidelines for physical activity during pregnancy recommend at least 150 min of moderate-intensity physical activity throughout the week, unless there is a medical reason to avoid physical activity [11, 12]. Although LTPA during pregnancy offers minimal risks and has been shown to benefit most women [12], unacceptably low levels of LTPA during pregnancy have been shown in multiple studies worldwide [13–15]. In this context, population-based studies that allow monitoring the progress of LTPA patterns over time and set goals for effective interventions to increase physical activity levels during pregnancy are extremely important.

In spite of the increasingly efforts to consistently monitor global physical activity patterns and the substantial progress made in surveillance in recent years [2], data on physical activity patterns for subgroups at high risk of inactivity in the population, such as pregnant women, are still missing. To date, only one study describing changes in physical activity patterns among pregnant women over time has been found in the worldwide literature [16]. Findings of this surveillance study carried out in the United States of America (USA) over an 8-year period (1999–2006) revealed no change in the proportion of pregnant women meeting the minimal recommendations for physical activity (21.6 vs. 24.3%). On the other hand, the proportion of women reporting any moderate LTPA practice increased from 46.8% in 1999–2002 to 58% in 2003–06.

In Pelotas, southern Brazil, 12.9% of the mothers from the 2004 Pelotas Birth Cohort Study reported to engage in any LTPA during pregnancy and only 4.3% reported continued LTPA during the whole pregnancy, regardless of frequency and weekly amount [13]. While there is a growing body of worldwide literature and accessible information regarding the benefits of physical activity during pregnancy in the last decade [17], no specific public strategy that could have influenced LTPA levels among pregnant women was implemented in the city at the population level. The aim of the present study is to describe changes in LTPA among Brazilian pregnant women over an 11-year period by comparing data from two Birth Cohort Studies carried out in 2004 and 2015. Changes of LTPA patterns according to maternal age, education, parity, income, pre-pregnancy Body Mass Index (BMI) and pre-pregnancy LTPA are also reported.

## Methods

### Research setting and study design

The present study analyzed data from two population-based birth cohort studies carried out in the city of Pelotas, Southern Brazil, in 2004 and 2015. Strategies to recruit participants were identical in both surveys. All five maternity hospitals (attending patients from both private and public insurance) located in Pelotas were visited daily from 1 January to 31 December of each year, and all births of mothers living in the urban areas of the city were identified (99% of deliveries are performed at hospitals). A total of 4244 and 4271 mothers were interviewed after delivery in 2004 and 2015, respectively. The non-response rate at recruitment was below 1.5% in both studies. Face-to-face interviews took place in the hospital within 24 h after the delivery. Trained interviewers collected information on mother-child health using a structured questionnaire. Interviews lasted 60 min, on average. A fieldwork supervisor repeated 10% of the interviews to check the quality of the information collected. Further methodological details of the 2004 study are available elsewhere [13, 18].

### Outcome measures and covariates

Type, frequency and average duration of sessions of LTPA in a typical week (7-day recall) were investigated in four time periods during the perinatal interview: the three-month period prior to pregnancy as well as the first, second and third trimesters of pregnancy. Up to three different physical activities were recorded for each period. Women were asked not to report commuting, household or occupational activities as LTPA. The instrument used to assess LTPA in 2015 was the same employed in 2004 [13]. The total LTPA score was generated by the sum of minutes per week spent on each activity. A cut-off point of 150 min per week was used to classify women as active or not in each period. We also explored trends in any LTPA practice by comparing the proportion of women who were engaged in LTPA regardless of weekly amount.

Maternal covariates assessed in the studies and used in the comparison analysis were maternal age, schooling, parity, household income, pre-pregnancy BMI, and pre-pregnancy LTPA. Maternal age was collected as a continuous variable and divided into four categories (13–19, 20–29, 30–39 and 40–47 years). Schooling was assessed as maternal years of formal education and categorized into four categories (0–4, 5–8, 9–11, 12 or more years). Family monthly income was assessed as the sum of incomes of household members in the past month and categorized into quintiles. Parity was categorized into three categories (1/2/≥3) according to the total number of live births, including the birth from the cohort. Pre-pregnancy BMI was categorized according to the World Health Organization criteria into underweight (<18.5 kg/m^2^), normal weight (18.5–24.9 kg/m^2^); overweigh (25.0–29.9 kg/m^2^) and obese (≥30 kg/m^2^) based on self-reported height and weight. Pre-pregnancy LTPA (≥150 min/week) was also considered as a covariate. Independent variables were identically collected in both surveys for comparability.

### Statistical analysis

Statistical analyses were carried out using Stata version 13.0 (StataCorp, College Station, TX, USA). Data analysis initially included the comparison of study populations in terms of sociodemographic, behavioral and health characteristics. Percentages and confidence intervals were used to compare the data between the studies. The prevalence of LTPA was estimated for each study, and its changes in the period were evaluated, including comparisons according to subgroups of the independent variables. The prevalence of each type of LTPA practiced was also compared between the studies. Chi-square test for difference in proportions across the study period was used. To identify correlates of LTPA in each period, the proportion of active women was described according to the subgroups of the independent variables and confidence intervals were calculated. In each year, logistic regression models were used to provide estimates for the adjusted odds ratios within each subgroup (with adjustment for the other variables studied). Associations between LTPA during pregnancy (outcome) and maternal age, income, schooling and parity were not adjusted for pre-pregnancy variables (BMI and LTPA) because they were considered mediators in the causal chain of its determination. Statistical significance was set at *p <* 0.05. We excluded from our analysis a group of 142 pregnant women from the 2015 Pelotas (Brazil) Birth Cohort Study who were randomly enrolled in the intervention group of a randomized controlled trial nested in the cohort to study the effects of an exercise program during pregnancy on mother and child health outcomes [19].

## Results

### Samples description

Data from a total of 4244 mothers in 2004 and 4129 mothers in 2015 were analysed in this study. Table 1 presents the mothers’ sociodemographic, behavior and health-related characteristics in both cohorts. The proportion of adolescent pregnancies decreased from 19.0% (95%CI 17.9–20.2) in 2004 to 15.0% (14.0–16.1) in 2015, while the proportion of mothers aged 30 to 39 years increased from 28.0% (95%CI 26.7–29.3) to 34.7% (95%CI 33.3–36.2). We observed an increase in the proportion of women with 12 or more years of formal education (9.9 to 30.0%). Employment during pregnancy increased from 40.1% (95%CI 38.6–41.5) to 55.5% (95%CI 58.5–61.4) during the period. A decrease in the proportion of women who reported being underweight before pregnancy from 7.4% (95%CI 6.5–8.4) in 2004 to 3.9% (95%CI 3.3–4.5) in 2015, accompanied by an increase in the proportion of overweight (20.1 to 27.4%) and obese (8.9 to 20.6%) was observed. Smoking during pregnancy significantly decreased between 2004 and 2015 (27.6 to 17.0%) while gestational diabetes nearly tripled (3.0 to 8.6%). Physical activity advice received from health professionals during prenatal care significantly decreased from 72.2% (95%CI 70.8–73.5) to 61.3% (95%CI 59.8–62.8) from 2004 to 2015. Over the period there was an increase in the median of family income from $206 in 2004 to $649 in 2015 (data not shown).

**Table 1:** Characteristics of the mothers in 2004 and 2015 Birth Cohort Studies. Pelotas, Brazil

Variables	2004 (*n =* 4244)		2015 (*n =* 4129)	
	*N*	% (95%CI)	*N*	% (95%CI)
Age (years)	*n = 4242*		*n = 4128*	
12 –19	848	19.0 (17.9 –20.2)	620	15.0 (14.0 –16.1)
20 –29	2224	49.8 (48.2 –51.2)	1950	47.3 (45.7 –48.8)
30 –39	1251	28.0 (26.7 –29.3)	1434	34.7 (33.3 –36.2)
40 –47	146	3.2 (2.8 –3.9)	124	3.0 (2.5 –3.6)
Skin Color	*n = 4192*		*n = 4122*	
White	2581	61.6 (60.1 –63.0)	2895	70.2 (68.8 –71.6)
Non-white	1611	38.4 (37.0 –39.9)	1227	29.8 (28.4 –31.2)
Schooling (years)	*n = 4202*		*n = 4127*	
0 –4	658	15.7 (14.6 –16.8)	390	9.5 (8.6 –10.4)
5 –8	1740	41.4 (39.9 –42.9)	1082	26.2 (24.9 –27.6)
9 –11	1385	33.0 (31.5 –34.4)	1415	34.3 (32.9 –35.7)
> =12	419	9.9 (9.1 –10.9)	1240	30.0 (28.7 –31.5)
Marital status	*n = 4244*		*n = 4128*	
Living with a partner	3542	83.5 (82.3 –84.5)	3524	85.4 (84.3 –86.4)
Living without a partner	702	16.5 (15.5 –17.7)	604	14.6 (13.6 –15.7)
Employement during pregnancy	*n = 4243*		*n = 4128*	
Yes	1700	40.1 (38.6 –41.5)	2270	55.0 (53.5 –56.5)
No	2543	59.9 (58.5 –61.4)	1858	45.0 (43.5 –46.5)
Pre-pregnancy BMI (Kg/m^2^)	*n = 2887*		*n = 4005*	
< 18.5	213	7.4 (6.5 –8.4)	155	3.9 (3.3 –4.5)
18.5 –24.9	1836	63.6 (61.8 –65.3)	1928	48.1 (46.6 –49.7)
25.0 –29.9	581	20.1 (18.7 –21.6)	1096	27.4 (26.0 –28.8)
> =30	257	8.9 (7.9 –10.0)	826	20.6 (19.4 –21.9)
Parity	*n = 4243*		*n = 4127*	
1 (primiparae)	1673	39.4 (38.0 –40.9)	2047	49.6 (48.1 –51.1)
2	1105	26.1 (24.7 –27.4)	1274	30.9 (29.5 –32.3)
3 or more	1465	34.5 (33.1 –36.0)	806	19.5 (18.3 –20.8)
Smoking during pregnancy^a^	*n = 4244*		*n = 4126*	
Yes	1172	27.6 (26.3 –29.0)	701	17.0 (15.9 –18.2)
No	3072	72.4 (71.0 –73.7)	3425	83.0 (81.8 –84.1)
Gestational diabetes mellitus^b^	*n = 4241*		*n = 4125*	
Yes	126	3.0 (2.5 –3.5)	353	8.6 (7.7 –9.5)
No	4115	97.0 (96.5 –97.5)	3772	91.4 (90.5 –92.3)
Gestational hypertension^b^	*n = 4236*		*n = 4126*	
Yes	1006	23.7 (22.5 –25.1)	1055	25.6 (24.3 –26.9)
No	3230	76.3 (74.9 –77.5)	3071	74.4 (73.1 –75.7)
Physical activity counselling during prenatal care	*n = 4155*		*n = 4029*	
Yes	2999	72.2 (70.8 –73.5)	2471	61.3 (59.8 –62.8)
No	1156	27.8 (26.5 –29.2)	1558	38.7 (37.2 –40.2)

^a^Smoking during all trimester of pregnancy

^b^Self-reported gestational diabetes mellitus and hypertension

### Prevalence and changes in LTPA during pregnancy

**Fig. 1:**
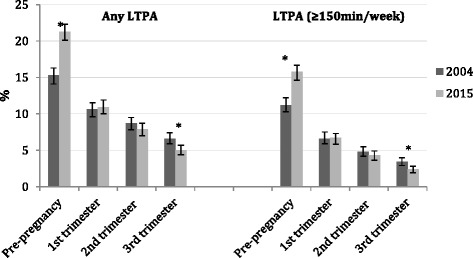
Changes in Leisure-time Physical Activity patterns before and during pregnancy. Pelotas, Brazil, 2004–2015. * Significant changes

Changes in LTPA patterns are presented in Fig. 1. There was an increase in the proportion of women practicing any pre-pregnancy LTPA between 2004 and 2015, from 15.3% (95%CI 14.2–16.3) to 21.3% (95%CI 20.1–22.6); as well as in the proportion of those engaged in recommended levels of LTPA, from 11.2% (95%CI 10.0–12.2) to 15.8% (95%CI 14.6–16.9). Regarding pregnancy LTPA patterns, no changes were observed for the first and second trimesters in the 11-year period. However, we observed a significant decrease in the prevalence of any LTPA from 6.6% (95%CI 5.9–7.5) to 3.4% (95%CI 2.9–4.0) as well as in recommended LTPA during the third trimester of pregnancy, declining from 5.0% (95%CI 4.3–5.7) to 2.4% (95%CI 1.9–2.8). In both cohort studies, the prevalence of LTPA (any or recommended) markedly declined from pre-pregnancy to the third gestational trimester (*p <* 0.001).

### Changes in patterns of LTPA practice during pregnancy

**Fig. 2:**
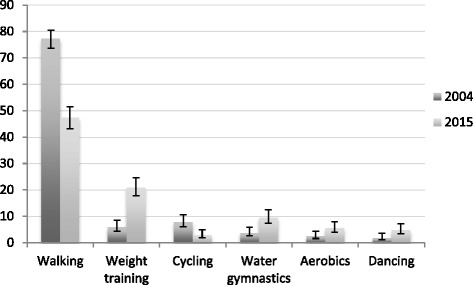
Changes in type of Leisure-Time Physical Activity practiced during pregnancy. Pelotas, Brazil, 2004–2015. * Among those practicing any LTPA during pregnancy (*N =* 562 in 2004; *N =* 549 in 2015). Only those activities with a significant change are presented

Among women engaged in LTPA during pregnancy, walking was the most reported type of LTPA in both surveys (Fig. 2). The second most commonly reported LTPA was cycling and weight training in 2004 and 2015, respectively. Regarding changes over time, reductions in walking (from 77.2% in 2004 to 47.4% in 2015) and cycling (from 8% in 2004 to 3.1% in 2015) and increases in weight training (from 6.1% in 2004 to 21% in 2015), water gymnastics (from 3.9% in 2004 to 9.7% in 2015), aerobics (from 2.7% in 2004 to 5.7% in 2015) and dancing (from 2% in 2004 to 4.9% in 2015) were observed.

### Changes in LTPA according to the subgroups of the independent variables

Tables 2 and 3 describe detailed time changes in the prevalence of recommended and any LTPA by maternal age, income, schooling, parity, pre-pregnancy BMI and pre-pregnancy LTPA from 2004 to 2015. The proportion of women considered active increased in the pre-pregnancy period among all groups of maternal age, income (except for the richest) and parity, from 2004 to 2015. Marked increases were also observed among those mothers with 5 to 8 years of formal education and those classified as underweight before the pregnancy. During the first and second trimesters of pregnancy, despite the overall stability in prevalence, decreases in recommended LTPA were observed for some subgroups of women. During the first gestational trimester, LTPA declined between 2004 and 2015 among women with 9 to 11 years of schooling and among those considered active before pregnancy. Decreases in the prevalence of recommended levels of LTPA in the second trimester of pregnancy were observed among mothers aged 20 to 29 years, classified in the intermediate quintile of family income, with at least 9 years of education, who were giving birth to the first child, obese and, among those engaged in recommended levels of LTPA before pregnancy. Declining changes for the third gestational trimester followed the same patterns observed for the second trimester of pregnancy, except that LTPA also declined among mothers belonging to the fourth quintile of family income and among those with a normal pre-pregnancy BMI.

**Table 2:** Changes in recommended LTPA (≥150 min/week) by maternal age, schooling, income, parity, pre-pregnancy BMI and pre-pregnancy LTPA. Pelotas, Brazil, 2004–2015

	Pre-pregnancy *N* (%)		% change	1st trimester *N* (%)		% change	2nd trimester *N* (%)		% change	3rd trimester *N* (%)		% change
Variables	2004	2015		2004	2015		2004	2015		2004	2015	
Maternal age (years)												
12 –19	7.8	12.3	+57.7**	5.1	5.2	+2.0	4.1	4.0	- 2.4	4.0	2.4	- 40.0
20 –29	210.4	14.9	+43.3**	6.3	5.3	- 15.9	5.0	3.1	- 38.0**	3.7	2.1	- 43.2**
30 –39	15.4	18.3	+18.8*	8.4	9.0	+7.1	5.6	5.9	+5.4	3.1	2.8	- 9.7
40 –47	8.5	16.9	+98.8*	4.3	8.9	+107.0	1.4	4.8	+242.9	1.4	2.4	+71.4
Family income (quintiles)												
1 (poorest)	5.3	11.7	+120.7**	3.8	3.5	- 17.9	2.5	2.9	+16.0	1.8	1.8	0
2	6.0	11.6	+93.3**	4.9	6.3	+28.6	3.5	4.0	+14.3	2.5	2.5	0
3	9.1	15.3	+68.1**	6.0	5.3	- 21.7	5.5	3.0	- 35.5*	4.0	2.2	- 35.0*
4	12.7	16.7	+31.5*	6.8	7.8	+14.7	4.7	3.4	- 27.7	3.4	1.5	- 55.9*
5 (wealthiest)	23.7	26.8	+13.1	11.7	11.8	+0.9	8.2	9.2	+12.2	5.6	4.7	- 16.1
Schooling (years)												
0 –4	4.3	5.6	+30.2	2.9	3.3	+13.8	2.0	2.6	+30.0	1.2	2.1	+75.0
5 –8	7.8	10.6	+35.9*	4.6	4.5	- 12.2	3.3	3.0	- 9.1	2.7	2.0	- 25.9
9 –11	13.4	14.3	+6.7	8.2	5.2	- 36.6**	5.7	3.1	- 45.6**	3.8	2.0	- 47.4**
≥ 12	27.2	25.1	- 17.7	14.6	11.5	- 21.2	11.9	7.3	- 18.0**	7.6	3.2	- 57.9**
Parity												
1	13.9	18.7	+34.5**	8.7	8.5	- 2.3	7.1	5.4	- 23.9*	5.1	2.9	- 43.2**
2	11.1	13.8	+24.3*	5.7	5.4	- 5.3	3.6	3.3	- 8.3	2.4	2.0	- 16.7
3 or more	8.3	11.4	+37.3*	4.8	4.2	- 12.5	3.1	2.9	- 6.5	2.2	1.6	- 27.3
Pre-pregnancy BMI												
< 18.5	5.6	11.6	+107.1*	4.2	5.8	+38.1	3.8	3.2	- 15.8	1.4	3.2	+128.6
18.5 –24.9	14.0	16.1	+15.0	7.8	7.2	- 17.7	6.1	5.2	- 14.8	4.6	2.8	- 39.1**
25.0 –29.9	14.3	17.8	+24.5	8.6	7.6	- 11.6	5.0	3.8	- 24.0	2.8	2.6	- 7.1
> =30	14.0	14.2	+1.4	8.2	5.3	- 35.4	6.2	3.3	- 46.8*	3.9	1.2	- 69.2*
Pre-pregnancy LTPA (≥150 min/week)												
Yes				47.7	34.8	- 27.0**	31.7	19.2	- 39.4**	19.3	9.7	- 49.7**
No				1.4	1.4	0	1.4	1.5	+7.1	1.4	1.0	- 28.6
Total	11.2	15.8	+41.1**	6.6	6.7	+1.5	4.8	4.3	- 10.4	3.4	2.4	- 29.4**

**X ^2^ for change 2004–2015 *p <* 0.01

*X ^2^ for change 2004–2015 *p <* 0.05

**Table 3:** Changes in any LTPA by maternal age, schooling, income, parity, pre-pregnancy BMI and pre-pregnancy LTPA. Pelotas, Brazil, 2004–2015

	Pre-pregnancy *N* (%)		% change	1st trimester *N* (%)		% change	2nd trimester *N* (%)		%change	3rd trimester *N* (%)		% change
Variables	2004	2015		2004	2015		2004	2015		2004	2015	
Maternal age (years)												
12 –19	11.3	16.6	+46.9**	9.1	8.2	- 9.9	7.8	6.5	- 16.7	7.9	4.4	- 44.3**
20 –29	14.2	19.2	+35.2**	10.3	9.0	- 12.6	8.9	16.0	- 32.6**	6.8	4.4	- 35.3**
30 –39	20.1	26.0	+29.4**	12.4	14.5	+16.9	9.6	11.1	+15.6	5.9	6.3	+6.8
40 –47	12.8	23.4	+82.8*	7.8	14.5	+85.9	4.3	7.3	+69.8	2.8	3.3	+17.9
Family income (quintiles)												
1 (poorest)	7.3	14.9	+104.1**	7.0	6.1	- 12.9	5.6	5.2	- 7.1	4.1	3.8	- 7.3
2	9.5	15.5	+63.2**	7.8	8.8	+12.8	5.2	5.9	+13.5	4.5	4.0	- 11.2
3	12.6	18.3	+45.2**	9.2	7.8	- 15.3	8.6	4.9	- 43.0**	7.1	3.6	- 49.3**
4	17.1	22.9	+33.9**	11.1	12.3	+10.8	9.0	6.9	- 23.3	7.1	3.8	- 46.8**
5 (wealthiest)	30.2	40.2	+33.1**	18.0	22.0	+22.2	15.2	18.7	+23.0	10.5	11.3	+7.6
Schooling (years)												
0-4	5.2	8.5	+63.5*	4.0	4.9	+22.5	2.7	4.1	+51.9	1.8	3.4	+88.9
5 –8	11.0	14.0	+27.3*	8.1	6.8	- 16.0	6.0	4.6	- 23.3	5.7	3.7	- 35.1*
9 –11	18.1	18.5	+2.2	12.9	8.2	- 36.4**	10.5	5.0	- 52.4**	7.4	3.4	- 54.1**
≥ 12	36.8	35.0	- 4.9	22.7	19.6	- 13.7	21.5	15.1	- 29.8**	13.6	8.5	- 62.5**
Parity												
1	19.4	25.5	+31.4**	14.1	13.8	- 2.1	13.0	10.1	- 32.3**	10.1	6.3	- 37.6**
2	14.3	19.3	+35.0**	9.7	9.7	0	7.0	6.5	- 7.1	5.3	4.2	- 20.8
3 or more	11.2	13.9	+24.1	7.2	5.7	- 20.8	5.1	4.4	- 13.7	3.6	2.9	- 19.5
Pre-pregnancy BMI												
< 18.5	9.4	15.5	+64.9	8.9	11.0	+23.6	6.6	7.1	+7.6	4.7	7.1	+51.1
18.5 –24.9	18.5	23.1	+24.9**	12.6	12.6	0	10.9	9.6	- 11.9	8.7	6.9	- 20.7*
25.0 –29.9	18.9	22.1	+16.9	13.1	11.0	- 16.0	8.6	7.0	- 18.6	5.6	4.1	- 26.8
> =30	8.7	18.8	+0.5	11.7	8.2	- 29.9	10.1	5.9	- 41.6*	5.8	2.1	- 63.8**
Pre-pregnancy LTPA (150 min/week)												
Yes				55.9	40.4	- 27.7**	41.2	24.9	- 39.6**	27.1	14.5	- 46.5**
No				4.8	5.4	+12.5	4.6	4.7	+2.2	4.0	3.2	- 20.0
Total	5.3	21.3	+39.2**	10.6	10.9	+2.8	8.7	7.9	- 9.2	6.6	5.0	- 16.7**

**X ^2^ for change 2004–2015 *p <* 0.01

*X ^2^ for change 2004–2015 *p <* 0.05

The proportion of women engaged in any LTPA before pregnancy also increased from 2004 to 2015 and among all subgroups of maternal age, income, schooling, parity and, pre-pregnancy LTPA (Table 3). In terms of pre-pregnancy BMI, marked increases in the prevalence of any LTPA were observed only among mothers classified as normal according to their pre-pregnancy BMI. During pregnancy the same patterns described for recommended LTPA were observed for any LTPA, except that in the third trimester of pregnancy it also decreased among adolescent mothers and those with intermediate schooling.

### Correlates of LTPA during pregnancy

Adjusted associations between recommended LTPA and the independent variables in 2004 and 2015 are presented in Table 4. During the pre-pregnancy period, mothers aged 30 to 39 years were more likely to reach recommended LTPA levels when compared to adolescent mothers in 2004 (OR 1.69; 95%CI 1.17–2.44) but no association between maternal age and pre-pregnancy LTPA was found in 2015. In both 2004 and 2015 studies, income and schooling were positively associated with pre-pregnancy recommended LTPA while parity was negatively associated. Pre-pregnancy LTPA was the strongest correlate of LTPA during pregnancy in all gestational trimesters and in both surveys. However, the magnitude of associations was smaller in 2015. During pregnancy, maternal age was associated with LTPA in the first trimester of pregnancy in 2004; with mothers aged 30 to 39 years being more likely to be engaged in recommended LTPA. In 2015, all confidence intervals included the null value. A significant positive association between recommended LTPA and income was identified only for the first trimester in the 2004 cohort. A positive association between recommended LTPA and schooling was observed in all trimesters in 2004, but in 2015 it was only observed for the first trimester. Parity was negatively associated with LTPA in all trimesters of pregnancy in both studies, except in the third trimester in 2015.

**Table 4:** Adjusted associations between recommended LTPA (150 min/week) and maternal characteristics across surveys. Pelotas, Brazil, 2004–2015

	Pre-pregnancy		1st trimester		2nd trimester		3rd trimester	
Variables	OR (95%CI)		OR (95%CI)		OR (95%CI)		OR (95%CI)	
	2004	2015	2004	2015	2004	2015	2004	2015
Maternal age (years)^a^								
	*p = 0.006*	*p = 0.636*	*p = 0.046*	*p = 0.013*	*p = 0.650*	*p = 0.106*	*p = 0.138*	*p = 0.554*
12 –19	1.0	1.0	1.0	1.0	1.0	1.0	1.0	1.0
20 –29	1.12 (0.81 –1.56)	0.94 (0.68 –1.30)	1.19 (0.80 –1.77)	0.93 (0.58 –1.49)	1.16 (0.74 –1.80)	0.70 (0.40 –1.22)	0.99 (0.61 –1.62)	0.95 (0.47 –1.90)
30 –39	1.69 (1.17 –2.44)	1.00 (0.70 –1.43)	1.69 (1.07 –2.67)	1.44 (0.85 –2.42)	1.34 (0.79 –2.27)	1.07 (0.57 –1.98)	0.71 (0.38 –1.34)	1.06 (0.48 –2.34)
40 –47	1.08 (0.54 –2.18)	1.07 (0.58 –1.96)	1.01 (0.40 –2.57)	1.68 (0.73 –3.86)	0.43 (0.10 –1.89)	1.13 (0.40 –3.15)	0.46 (0.10 –2.09)	1.11 (0.28 –4.44)
Family income (quintiles)^a^	*p < 0.001*	*p = 0.05*	*p = 0.017*	*p = 0.061*	*p = 0.119*	*p = 0.073*	*p = 0.110*	*p = 0.068*
1 (poorest)	1.0	1.0	1.0	1.0	1.0	1.0	1.0	1.0
2	1.21 (0.80 –1.84)	0.87 (0.63 –1.19)	1.32 (0.83 –2.11)	1.71 (1.06 –2.77)	1.45 (0.83 –2.55)	1.33 (0.76 –2.31)	1.39 (0.72 –2.69)	1.45 (0.72 –2.94)
3	1.64 (1.11 –2.43)	1.04 (0.77 –1.42)	1.37 (0.87 –2.17)	1.26 (0.76 –2.10)	1.93 (1.14 –3.28)	0.91 (0.49 –1.67)	2.05 (1.11 –3.78)	1.32 (0.63 –2.77)
4	2.10 (1.44 –3.07)	0.95 (0.70 –1.29)	1.36 (0.86 –2.14)	1.55 (0.95 –2.53)	1.42 (0.82 –2.47)	0.87 (0.47 –1.59)	1.59 (0.84 –3.03)	0.87 (0.39 –1.97)
5 (wealthiest)	3.23 (2.21 –4.73)	1.36 (0.98 –1.90)	1.80 (1.14 –2.85)	1.75 (1.04 –2.95)	1.70 (0.98 –2.95)	1.88 (1.03 –3.43)	1.80 (0.94 –3.46)	2.64 (1.21 –5.78)
Schooling (years)^a^								
	*p < 0.001*	*p < 0.001*	*p < 0.001*	*p = 0.006*	*p < 0.001*	*p = 0.073*	*p < 0.001*	*p = 0.583*
0 –4	1.0	1.0	1.0	1.0	1.0	1.0	1.0	1.0
5 –8	1.65 (1.08 –2.52)	2.07 (1.24 –3.43)	1.50 (0.89 –2.52)	1.23 (0.64 –2.37)	1.51 (0.74 –1.80)	1.11 (0.52 –2.38)	1.85 (0.86 –3.98)	0.84 (0.37 –1.94)
9 –11	2.09 (1.35 –3.25)	2.72 (1.65 –4.50)	2.23 (1.31 –3.82)	1.23 (0.64 –2.36)	2.18 (1.15 –4.12)	1.16 (0.54 –2.49)	2.23 (1.00 –4.95)	0.72 (0.31 –1.69)
≥ 12	3.46 (2.12 –5.66)	4.74 (2.80 –8.03)	3.33 (1.82 –6.11)	2.20 (1.11 –4.35)	4.42 (2.17 –8.99)	1.97 (0.87 –4.45)	4.77 (1.97 –11.53)	0.77 (0.28 –1.98)
Parity^a^								
	*p = 0.004*	*p = 0.02*	*p = 0.002*	*p = 0.001*	*p = 0.003*	*p = 0.012*	*p = 0.040*	*p = 0.090*
1	1.0	1.0	1.0	1.0	1.0	1.0	1.0	1.0
2	0.74 (0.57 –0.95)	0.75 (0.61 –0.93)	0.57 (0.41 –0.80)	0.62 (0.45 –0.84)	0.50 (0.34 –0.74)	0.60 (0.40 –0.89)	0.51 (0.32 –0.82)	0.73 (0.44 –1.21)
3	0.66 (0.49 –0.88)	0.80 (0.60 –1.07)	0.59 (0.41 –0.84)	0.55 (0.35 –0.86)	0.56 (0.37 –0.85)	0.66 (0.39 –1.14)	0.62 (0.38 –1.03)	0.61 (0.30 –1.24)
Pre-pregnancy BMI								
	*p = 0.713*	*p = 0.423*	*p = 0.399*	*p = 0.485*	*p = 0.783*	*p = 0.056*	*p = 0.198*	*p = 0.198*
< 18.5	0.40 (0.21 –0.76)	0.90 (0.53 –1.51)	1.00 (0.43 –2.34)	1.26 (0.56 –2.86)	1.04 (0.45 –2.38)	0.83 (0.31 –2.21)	0.40 (0.12 –1.35)	1.35 (0.51 –3.62)
18.5 –24.9	1.0	1.0	1.0	1.0	1.0	1.0	1.0	1.0
25.0 –29.9	1.06 (0.80 –1.41)	1.24 (1.00 –1.53)	1.23 (0.80 –1.89)	1.02 (0.72 –1.44)	0.82 (0.51 –1.33)	0.70 (0.47 –1.06)	0.61 (0.34 –1.10)	0.83 (0.51 –1.37)
> =30	1.11 (0.75 –1.65)	0.99 (0.77 –1.26)	1.14 (0.61 –2.10)	0.81 (0.53 –1.23)	1.11 (0.58 –2.10)	0.70 (0.43 –1.13)	0.94 (0.45 –1.96)	0.44 (0.21 –0.92)
Pre-pregnancy LTPA (≥150 min/week)			*p < 0.001*	*p < 0.001*	*p < 0.001*	*p < 0.001*	*p < 0.001*	*p < 0.001*
Yes			51.7 (35.0 –76.4)	37.8 (26.5 –53.8)	26.9 (18.0 –40.1)	16.2 (11.2 –23.5)	15.6 (10.0 –24.2)	11.8 (7.4 –18.7)
No			1.0	1.0	1.0	1.0	1.0	1.0

^a^Adjusted for all other variables except pre-pregnancy BMI and LTPA

In terms of any LTPA, similar patterns of associations were observed for all independent variables (Table 5). However, positive associations with income were observed in all pregnancy trimesters in 2015 and negative associations with parity were observed in all periods for both studies. Besides, negative associations between pre-pregnancy BMI and any LTPA were observed for all pregnancy trimesters in 2015.

**Table 5:** Adjusted associations between any LTPA and maternal characteristics across surveys. Pelotas, Brazil, 2004–2015

	Pre-pregnancy		1st trimester		2nd trimester		3rd trimester	
Variables	OR (95%CI)		OR (95%CI)		OR (95%CI)		OR (95%CI)	
	2004	2015	2004	2015	2004	2015	2004	2015
Maternal age (years)^a^								
	*p = 0.003*	*p = 0.088*	*p = 0.074*	*p = 0.004*	*p = 0.499*	*p = 0.076*	*p = 0.052*	*p = 0.599*
12 –19	1.0	1.0	1.0	1.0	1.0	1.0	1.0	1.0
20 –29	1.11 (0.84 –1.48)	0.92 (0.70 –1.23)	1.14 (0.84 –1.55)	0.89 (0.61 –1.31)	1.09 (0.78 –1.52)	0.72 (0.46 –1.12)	0.85 (0.60 –1.21)	0.91 (0.55 –1.53)
30 –39	1.64 (1.19 –2.27)	1.11 (0.81 –1.53)	1.47 (1.02 –2.12)	1.29 (0.84 –1.96)	1.27 (0.85 –1.89)	1.03 (0.63 –1.67)	0.73 (0.47 –1.14)	1.02 (0.57 –1.82)
40 –47	1.21 (0.67 –2.20)	1.17 (0.68 –2.02)	1.11 (0.54 –2.26)	1.62 (0.82 –3.18)	0.72 (0.29 –1.79)	0.84 (0.36 –1.95)	0.47 (0.16 –1.39)	0.63 (0.19 –1.99)
Family income (quintiles)^a^	*p < 0.001*	*p = <0.001*	*p = 0.018*	*p = <0.001*	*p = 0.045*	*p = 0.001*	*p = 0.023*	*p = 0.002*
1 (poorest)	1.0	1.0	1.0	1.0	1.0	1.0	1.0	1.0
2	1.34 (0.95 –1.89)	0.92 (0.70 –1.22)	1.13 (0.79 –1.63)	1.38 (0.93 –2.04)	0.94 (0.61 –1.43)	1.07 (0.69 –1.67)	1.11 (0.70 –1.78)	1.07 (0.63 –1.83)
3	1.56 (1.12 –2.18)	0.98 (0.74 –1.30)	1.15 (0.81 –1.65)	1.06 (0.70 –1.59)	1.35 (0.92 –1.99)	0.79 (0.49 –1.27)	1.62 (1.05 –2.51)	0.93 (0.54 –1.62)
4	1.94 (1.40 –2.68)	1.07 (0.81 –1.41)	1.23 (0.87 –1.76)	1.42 (0.97 –2.10)	1.22 (0.82 –1.80)	0.90 (0.57 –1.42)	1.54 (0.99 –2.40)	0.87 (0.50 –1.51)
5 (wealthiest)	2.72 (1.96 –3.78)	1.82 (1.35 –2.45)	1.56 (1.09 –2.24)	2.04 (1.35 –3.07)	1.43 (0.96 –2.12)	1.97 (1.25 –3.09)	1.64 (1.04 –2.59)	2.29 (1.33 –3.95)
Schooling (years)^a^								
	*p = <0.001*	*p = <0.001*	*p = <0.001*	*p = <0.001*	*p < 0.001*	*p < 0.001*	*p < 0.001*	*p = 0.242*
0 –4	1.0	1.0	1.0	1.0	1.0	1.0	1.0	1.0
5 –8	2.00 (1.37 –2.95)	1.71 (1.12 –2.61)	1.94 (1.25 –2.99)	1.20 (0.70 –2.07)	1.95 (1.16 –3.26)	0.99 (0.54 –1.82)	2.54 (1.37 –4.70)	0.94 (0.49 –1.79)
9 –11	2.63 (1.77 –3.92)	2.17 (1.42 –3.30)	2.62 (1.67 –4.12)	1.25 (0.73 –2.14)	2.82 (1.66 –4.79)	1.09 (0.60 –1.99)	2.80 (1.48 –5.30)	0.77 (0.40 –1.50)
≥ 12	5.06 (3.25 –7.90)	3.86 (2.47 –6.00)	4.25 (2.55 –7.07)	2.37 (1.35 –4.16)	5.80 (3.24 –10.38)	2.39 (1.27 –4.49)	5.45 (2.71 –10.98)	1.30 (0.64 –2.65)
Parity^a^								
	*p < 0.001*	*p < 0.001*	*p < 0.001*	*p < 0.001*	*p < 0.001*	*p = 0.001*	*p < 0.001*	*p = 0.012*
1	1.0	1.0	1.0	1.0	1.0	1.0	1.0	1.0
2	0.65 (0.52 –0.82)	0.73 (0.60 –0.88)	0.62 (0.47 –0.80)	0.67 (0.52 –0.86)	0.51 (0.38 –0.69)	0.65 (0.49 –0.87)	0.57 (0.41 –0.79)	0.73 (0.52 –1.04)
3	0.63 (0.49 –0.81)	0.66 (0.51 –0.87)	0.55 (0.41 –0.74)	0.49 (0.33 –0.71)	0.48 (0.34 –0.67)	0.60 (0.39 –0.92)	0.51 (0.35 –0.75)	0.59 (0.35 –1.00)
Pre-pregnancy BMI								
	*p = 0.517*	*p = 0.581*	*p = 0.566*	*p = 0.043*	*p = 0.715*	*p = 0.021*	*p = 0.110*	*p < 0.001*
< 18.5	0.51 (0.31 –0.83)	0.82 (0.51 –1.31)	1.08 (0.62 –1.88)	1.19 (0.65 –2.17)	0.82 (0.45 –1.51)	0.90 (0.45 –1.81)	0.61 (0.31 –1.21)	1.19 (0.59 –2.39)
18.5 –24.9	1.0	1.0	1.0	1.0	1.0	1.0	1.0	1.0
25.0 –29.9	1.08 (0.84 –1.39)	1.05 (0.87 –1.27)	1.13 (0.81 –1.58)	0.88 (0.68 –1.16)	0.83 (0.57 –1.20)	0.75 (0.55 –1.02)	0.73 (0.49 –1.10)	0.60 (0.42 –0.87)
> =30	1.15 (0.81 –1.64)	0.89 (0.72 –1.11)	1.04 (0.64 –1.69)	0.70 (0.51 –0.97)	1.13 (0.69 –1.84)	0.71 (0.49 –1.01)	0.81 (0.45 –1.45)	0.33 (0.19 –0.56)
Pre-pregnancy LTPA (≥150 min/week)			*p < 0.001*	p < 0.001	*p < 0.001*	*p < 0.001*	*p < 0.001*	*p < 0.001*
Yes			19.1 (14.4 –25.3)	10.3 (8.19 –13.0)	11.6 (8.67 –15.5)	5.63 (4.36 –7.28)	7.25 (5.27 –9.97)	4.45 (3.26 –6.07)
No			1.0	1.0	1.0	1.0	1.0	1.0

^a^Adjusted for all other variables except pre-pregnancy BMI and LTPA

## Discussion

The present study compared LTPA levels prior to and during pregnancy among Brazilian women over an 11-year period (2004–2015). The findings indicated an increase in the proportion of women engaged in pre-pregnancy LTPA over the period. On the other hand, no change in LTPA levels was observed for the first and second trimesters of pregnancy while a decline was observed for the last trimester from 2004 to 2015. Overall, major decreases in the prevalence of LTPA were observed among young mothers (20 to 29 years), classified in the third and fourth quintiles of family income, with higher schooling (≥9 years of formal education), first-time mothers, obese according to their pre-pregnancy BMI and who were engaged in recommended levels of LTPA before pregnancy. Although the total prevalence of LTPA remained unchanged in 2015 for the first and second trimesters of pregnancy as compared to 2004, declines in LTPA patterns were observed for different subgroups of the population, which followed very similar patterns observed for the third trimester of pregnancy.

The increase in the number of women engaged in pre-pregnancy LTPA observed in the most recent cohort study is in line with LTPA trend patterns described for Brazilian adults based on recent data from a national surveillance system [20]. From 2006 to 2012, the prevalence of recommended LTPA increased from 12.8% to 14.9% among the adult Brazilian population. During this period, a marked increase was observed particularly among young adults, which seems to encompass the population of women of childbearing age who are part of our cohort. On the other hand, our findings showed that pre-pregnancy LTPA has become a less important predictor of pregnancy LTPA over the period, with a greater number of previously active women discontinuing or decreasing LTPA engagement during pregnancy in 2015, as compared with 2004.

National efforts to promote physical activity were recently intensified in the country in an attempt to decrease the burden of non-communicable diseases and this may partly explain the increase in pre-pregnancy LTPA levels observed [21]. Nevertheless, as we can observe through our findings, the possible increased population awareness about LTPA benefits does not seem to be translated into practice when it comes to the pregnancy period. This is particularly relevant because inactivity during pregnancy is known to be associated with increased risks of maternal complications [5], also influencing maternal long-term chronic disease risk and susceptibility in the offspring [10, 22]. Therefore, as inactivity during pregnancy tends to remain in the postpartum period and beyond [3, 4], the implementation of physical activity interventions that target pregnant women may positively influence future overall physical activity trends and might be considered by policy makers as part of a strategy to effectively reduce the burden of non-communicable diseases at the population level.

Data from the USA for 1999–2006 on national physical activity trends among pregnant women had shown that participation in any moderate LTPA increased about 24% over the 7-year period (from 46.8% in 1999 to 58.0% in 2006) [16]. At the same time, the proportion of women meeting recommendations for LTPA remained stable [16]. Whilst the reference period assessed by the authors does not allow for a direct comparison with our findings, the increasing trend in any LTPA patterns reported among USA pregnant women goes in the opposite direction of a decrease in both any and recommended levels of LTPA during pregnancy found in the present study. Besides, the proportion of women engaged in LTPA reported by the authors was much higher than estimates found in the current study among Brazilian pregnant women.

To compare our data, however, we need to consider that research on physical activity during pregnancy and its influence on maternal-child health outcomes started at least three decades ago in high-income countries and LTPA promotion among pregnant women seems to have been an issue of concern for some time [23]. While the American College of Obstetricians and Gynecologists guidelines for physical activity during pregnancy were initially released in 1985 and have been discussed and reaffirmed over time, in Brazil, recommendations to provide guidance to health care providers are still nonexistent; the available scientific knowledge regarding physical activity during pregnancy seems not being translated to clinical practice and adopted by the population.

A similar pattern of disparity in physical activity promotion progress can be described if we take a look at trends in LTPA participation among the adult population worldwide. While increases in LTPA patterns have been reported for adults living in high-income countries in the past 20–30 years [24], in Brazil they recently began to be observed. In this sense, it is possible that advances made in LTPA patterns among the Brazilian adult population are too recent to have also impacted LTPA patterns during pregnancy given that interventions to target specifically pregnant women are still missing in the country. Moreover, LTPA participation during pregnancy is a more complex behavior to modify once it is often surrounded by misconceptions and uncertainty regarding its benefits to the mother-child health as well as the symptoms and limitations of the gestational period [25].

Among the barriers preventing women to engage in LTPA practice during pregnancy, the lack of knowledge to make decisions about exercise is one of the most reported in the literature [25]. In this context, being the most influent source of information for pregnant women, healthcare providers play an important role in providing pregnant women with the necessary knowledge and support to engage in antenatal physical activity [26]. However, even in high income countries where LTPA during pregnancy is more frequently promoted, results from a study had shown that over a third of health professionals were not confident in their knowledge regarding benefits and risks of LTPA during pregnancy [27]. Importantly, in the present study counseling made by prenatal healthcare professionals on LTPA practice declined over time. Therefore, there is an urgent need to increase awareness of physical activity benefits during pregnancy and the available recommendations among healthcare providers. In this context, implementation and dissemination research is and essential framework to further understand the gap between knowledge and practice [28] related to LTPA promotion during pregnancy [29].

Intriguingly, in our study a pattern of decline in pregnancy LTPA levels over the period was particularly observed for those mothers belonging to the groups of high income and education. Since healthy behaviors tend to be first adopted by those who have greater access to information, education and economic resources for prevention, an opposite trend would be expected compared to what we observed [30]. Smoking patterns among pregnant women in Brazil, for example, has been declining markedly since the 80’s, with greater and faster declines being observed among women with higher income [31]. However, tobacco-control policies are more advanced in the country compared to physical activity promotion, and the increased awareness about the harmful effects of smoking during pregnancy seems to have been translated into a favorable change of behavior by the population. In the present study smoking during pregnancy decreased 37% in this 11-year period.

To better understand the changes observed in the present study we are unable to dissociate the marked changes in socioeconomic, demographic and health characteristics during the time period assessed. The observed improvement in income and schooling was accompanied by an increase in maternal obesity and gestational diabetes that is certainly playing a role in LTPA changes observed and may also suggest a population undergoing a nutrition transition. The lower LTPA during pregnancy among pre-pregnancy obese women in 2015, especially in the last gestational trimester, are in agreement with a recent study showing decreased levels of exercise as pregnancy advances, particularly among pre-pregnancy obese women [32]. Besides, maternal obesity itself is associated with a higher risk of clinical complications such as gestational diabetes, hypertension and preeclampsia [33–35]. Accordingly, in the 2015 cohort study the prevalence of gestational diabetes was 17% among pre-pregnancy obese women compared to 4.1% among women with normal BMI, while the prevalence of hypertension was 45.9% and 16.8%, respectively (data not shown).

From 2004 to 2015, a significant decrease in LTPA patterns was also observed among obese women and this was especially evident in the last trimester of pregnancy, which might be related to an increase in the severity of maternal complications over time. In this sense, it is also possible that the increase trend in obesity and its related health complications had imposed challenges in the prenatal care assistance, having a negative impact in the confidence to counseling LTPA among healthcare providers. Although LTPA participation during pregnancy had been shown to have benefits in the prevention and/or management of gestational diabetes mellitus, excessive gestational weight gain and hypertensive disorders [5, 36–38], a clear disconnection between scientific evidence and clinical practice exists and the lack of knowledge of risks and benefits of LTPA during pregnancy is very common [29]. Furthermore, prenatal healthcare providers perceived barriers have been shown to increase when providing antenatal counseling for obese women [39].

Regarding correlates of LTPA during pregnancy, overall our findings support previous studies reporting positive associations between LTPA with maternal education, income and pre-pregnancy LTPA as well as a negative association with parity in both time points [40, 41]. However, suitable changes in the shape of associations could be observed over the period. While in 2004 the associations between LTPA and schooling was clearly linear, in 2015 only women from the highest schooling category had a greater probability of being active compared to women in the reference group (0–4 years of formal education). Moreover, although pre-pregnancy LTPA was the strongest predictor of LTPA engagement during pregnancy in both cohorts, the strength of the association decreased from 2004 to 2015.

Similar to other studies reporting on type of LTPA among pregnant women [42, 43], walking was the most prevalent LTPA during pregnancy in both cohorts. However, a significant decrease in the proportion of pregnant women engaged in walking was observed from 2004 to 2015 while other activities such as weight training, water gymnastics, aerobics and dancing increased. This shift in the type of LTPA practiced during pregnancy might reflect important changes in women’s beliefs and preferences over time. It might be possible that pregnant women engaged in LTPA nowadays are enough confident about its benefits and, therefore, feeling comfortable to explore a broader range of activities that used to be avoided in the past due to safety concerns and lack of knowledge. Changes in the type of LTPA performed over time with an increase in gym-related activities have been reported in monitoring studies conducted with other populations and may also be reflecting a generational effect [44].

### Strengths and limitations

The similarity of data collection methods between both surveys combined with the high response rates, are certainly the major strengths of the present study. The use of the same inclusion criteria and methodologies over an 11-year period provides reliable findings. Besides, LTPA patterns were measured during the different trimesters of pregnancy allowing the distinction of specific changes that happened across gestational trimesters.

Some limitations of the study need to be taken into consideration while interpreting the findings. First, instruments based on self-report such as the one used in this study, might overestimate physical activity levels. However, as this limitation was equally present in both surveys, comparability over time was not impaired. Second, the intensity of the reported leisure-time physical activities was not measured and for this reason we could not assess its changes over time. Yet, the lack of information on intensity was purposeful given that intensity prompts (amount of moderate or vigorous-intensity activities) would not be ideal in a retrospective analysis—women might remember how much they practiced some months ago, but probably not the intensity of the activities performed. Third, the retrospective assessment of LTPA could result in recall bias. But in the worst scenario mothers had to report their LTPA patterns 9 months before the interview (pre-pregnancy LTPA), which is considered a reasonable period since long-term maternal recall of pregnancy-related events has been suggested to be highly accurate [45]. Lastly, it should also be noted that the prevalence estimates reported in the present study might have being influenced by the fact that we excluded 141 mothers from the 2015 Birth Cohort Study who took part in an exercise intervention during pregnancy and who were not reaching recommended levels of LTPA during recruitment (inclusion criteria in the trial). Nevertheless, given the low probability of previously inactive women starting to practice physical activity during the pregnancy period [46] we expect a slight overestimation in the observed prevalence of LTPA during pregnancy in 2015. Consequently, the decline trends observed could be worse if including this sample of women.

## Conclusions and recommendations

The present study contributed to fill the knowledge gap on population LTPA trends during pregnancy. Our findings showed a significant increase in the proportion of women engaged in LTPA prior to pregnancy. On the other hand, no improvements in LTPA levels during pregnancy were observed over the 11-year period assessed, whereas a significantly decrease was observed for the third trimester of pregnancy. Future research might still include assessment of mother-child health benefits of physical activity during pregnancy, but must also address challenges in terms of implementation and dissemination of physical activity promotion. Moving towards to action, there is pressing need for intervention strategies aimed at increasing LTPA levels among pregnant women to reverse this trend and help stall the progression of its negative health consequences. Women should be encouraged to view the preconception period and pregnancy as opportunities to adopt healthy behaviors such as LTPA that could be maintained throughout life. In this context, healthcare providers involved in prenatal care play an essential role in advising women on LTPA benefits during pregnancy and encouraging them to start or continue exercising.

## Abbreviations

LTPA: Leisure-time physical activity
BMI: Body Mass Index
